# Baseline Alpha-Fetoprotein, Alpha-Fetoprotein-L3, and Des-Gamma-Carboxy Prothrombin Biomarker Status in Bridge to Liver Transplant Outcomes for Hepatocellular Carcinoma

**DOI:** 10.3390/cancers13194765

**Published:** 2021-09-23

**Authors:** Kelley G. Núñez, Tyler Sandow, Daniel Fort, Jai Patel, Mina Hibino, Ian Carmody, Ari J. Cohen, Paul Thevenot

**Affiliations:** 1 Institute of Translational Research, Ochsner Health, New Orleans, LA 70121, USA; Kelley.nunez@ochsner.org (K.G.N.); jai.patel@ochsner.org (J.P.); mina.hibino@ochsner.org (M.H.); acohen@ochsner.org (A.J.C.); 2 Department of Radiology, Ochsner Health, New Orleans, LA 70121, USA; tyler.sandow@ochsner.org; 3 Center for Outcomes and Health Services Research, Ochsner Health, New Orleans, LA 70121, USA; Daniel.fort@ochsner.org; 4 Multi-Organ Transplant Institute, Ochsner Health, New Orleans, LA 70121, USA; icarmody@ochsner.org; 5 Faculty of Medicine, The University of Queensland, New Orleans, LA 70121, USA

**Keywords:** alpha fetoprotein, AFP-L3, des-γ-carboxy prothrombin, hepatocellular carcinoma, liver-directed therapy

## Abstract

**Simple Summary:**

An abnormal alpha-fetoprotein (AFP) test is often associated with hepatocellular carcinoma (HCC) development, although as many as 40% of HCC diagnoses are made in the absence of an abnormal AFP test. In Japan and other Asian countries, Lens culinaris agglutinin-reactive AFP fraction (AFP-L3) and des-gamma-carboxy prothrombin (DCP) are used in combination with AFP for HCC diagnosis. Combined testing with all three biomarkers increases early diagnosis in addition to providing a patient-specific profile of HCC aggressiveness. The utility of AFP, AFP-L3, and DCP for HCC prognosis in the bridge to liver transplantation has not been established. The goal of this study is to define prognosis to first-line HCC treatment and the risk of progression prior to liver transplantation associated with biomarker profile at diagnosis. Biomarker profiling may have future implications in precision therapeutic management of HCC as a bridge to transplantation.

**Abstract:**

The biomarkers α-fetoprotein (AFP), Lens culinaris agglutinin-reactive AFP fraction (AFP-L3), and des-γ-carboxy prothrombin (DCP) have emerging implications in hepatocellular carcinoma (HCC) surveillance, overall prognosis, and post-surgical recurrence risk. This retrospective study investigated treatment and bridge to liver transplant (LT) prognosis associated with AFP, AFP-L3%, and DCP biomarker profiles prior to liver-directed therapy (LDT). In a 140-patient cohort, each biomarker was associated with HCC progression risk using the established thresholds of AFP > 20 ng/mL, AFP-L3 > 15%, and DCP > 7.5 ng/mL. Over 60% of the cohort expressed at least one biomarker at baseline. Although most biomarker-positive patients expressed the clinical standard AFP (57/87), only 32% were positive for AFP alone. Biomarker accumulation increased HCC progression risk but was not associated with demographic factors or preserved liver function. Biomarker triple negative patients had smaller index HCC (*p* = 0.003), decreased multifocal burden (*p* = 0.010), and a higher objective response rate (ORR, 62% compared to 46%, *p* = 0.011). Expressing all three biomarkers at baseline was associated with dismal first-line ORR (12%) with a median time to progression (TTP) of only 181 days post-LDT. Patients with triple negative status for the HCC biomarkers AFP, AFP-L3%, and DCP have the highest first-line ORR with < 5% HCC progression 1-year post-LDT. Biomarker profiling can establish baseline prognosis for identifying optimal bridge to LT and downstaging to LT candidates with triple negative biomarker status and providing an ideal post-LDT target as a compliment to radiographic response.

## 1. Introduction

Hepatocellular carcinoma (HCC) is a leading cause of cancer-related deaths. In patients with end-stage liver disease due to cirrhosis, liver transplantation (LT) provides the only curative treatment option to treat both the underlying disease and malignancy [1]. Alpha-fetoprotein (AFP) is the most widely utilized biomarker for HCC surveillance and monitoring response to liver-directed therapy (LDT). The AFP expression level has been linked to HCC progression risk, waitlist and overall survival, and recurrence-free survival after the resection of LT. With active HCC surveillance in patients with cirrhosis, AFP levels at diagnosis continue to trend lower, with many recent studies reporting interquartile ranges of AFP < 20 ng/mL [2,3]. Declining baseline AFP levels after the diagnosis of early-stage HCC have led to a concomitant decline in the threshold levels associated with the risk of HCC progression (AFP > 8–20 ng/mL) [4]. Although historical AFP levels are still prevalent in the literature (> 100 ng/mL [5] and > 400 ng/mL [6]), their ability to assess prognosis in the current landscape of early-stage HCC is limited. With AFP-based risk thresholds in early-stage HCC approaching the upper levels of normal (8 ng/mL), there is a need for new biomarkers associated with aggressive HCC biology and progression risk following LDT.

Lens culinaris agglutinin-reactive AFP fraction (AFP-L3) and des-γ-carboxy prothrombin (DCP) have emerged as biomarker candidates that increase the sensitivity for detecting HCC in combination with AFP. AFP-L3 is a fucosylated glycoform of AFP produced exclusively by malignant liver cells. DCP is a prothrombin variant that lacks post-translational carboxylation of glutamate residues due to impaired carboxylase activity in HCC. In Japan and other parts of Asia, AFP-L3 and DCP have been utilized for decades in the surveillance and management of HCC [7,8]. In expansive retrospective analysis, studies from these regions have validated AFP, AFP-L3, and DCP levels as risk factors for recurrence post-resection as well as recurrence-free survival outcomes across the spectrum of non-surgical, locally advanced, and intermediate to advanced HCC [9,10,11,12,13,14,15,16,17]. More recently, the AFP, AFP-L3, and DCP panel was validated as an effective strategy to assess HCC recurrence risk in the preoperative setting before LT [18,19].

Although AFP, AFP-L3, and DCP profiles have been intimately linked with aggressive HCC biology over the past three decades, most of these studies have focused on very early (BCLC-0), resectable HCC and palliative management of intermediate–advanced (BCLC C-D) HCC. The utility of AFP, AFP-L3, and DCP in LT for HCC has not been established [20], and recommendations for their application in HCC management are absent in the 2018 guidelines of the American Association for the Study of Liver Diseases [21]. Currently, AFP remains the clinical standard biomarker for assessing biological aggressiveness and the risk of disease progression in bridge to LT, early-stage HCC.

To address the role of baseline biomarker expression and LDT outcomes in early-stage HCC, we prospectively enrolled newly diagnosed patients scheduled to receive LDT as a bridge to LT. Baseline AFP, AFP-L3, and DCP levels were measured prior to LDT with patients analyzed according to their cumulative expression of the biomarkers. Baseline factors associated with the accumulation of biomarkers were investigated along with the role of the biomarkers in the objective response rate [1] to LDT and HCC progression risk.

## 2. Materials and Methods

### 2.1. Study Design, Setting, and Participants

**Figure 1:**
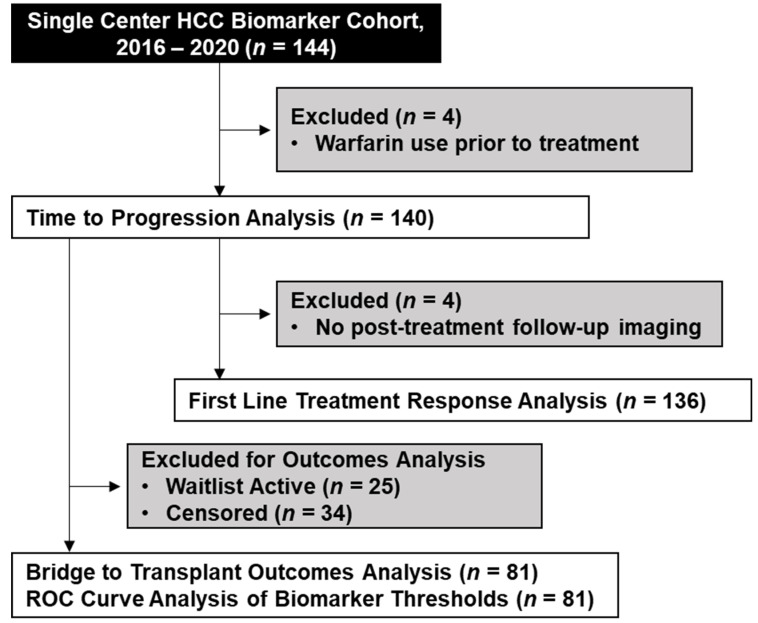
CONSORT Diagram of the Study Cohort. Outline of a single center retrospective analysis of prospectively collected data to assess time to progression and outcomes associated with expression of the AFP, AFP-L3, and DCP biomarkers.

This study was conducted in accordance with the ethical guidelines set forth by the Declaration of Helsinki. This study was approved by the Ochsner Health Institutional Review Board (study number 2016.131.B). Informed consent was obtained from patients participating in the observational prospective biomarker cohort study. An initial pilot cohort of patients with baseline biomarker data, defined as a binary variable of 0–1 or 2–3 positive biomarkers, and reaching primary endpoint of HCC progression or LT (*n* = 34) was utilized to power the current study. Using a dichotomous endpoint and two independent sample analyses at alpha 0.05 and beta 0.05, a sample size of *n* = 33 was obtained. The study was designed as a single-center, prospective study in patients receiving first-line LDT at a tertiary liver transplant center (Ochsner Multi-Organ Transplant Institute, New Orleans, LA, USA). The Ochsner Multi-Organ Transplant Institute is a high volume, short wait-time center. Inclusion criteria were a diagnosis of HCC based on the Liver Imaging Reporting and Data System (LI-RADS) and/or biopsy with a pending appointment to receive LDT as a bridge or downstage to LT in accordance with current American Association for the Study of Liver Disease guidelines [21]. Informed consent was obtained from patients prior to receiving LDT, with enrollment dates between August 2016 and October 2020. Patients on anticoagulant therapy prior to LDT were retroactively excluded from data analysis due to therapy-linked elevations in DCP. A breakdown of the cohorts used in this study is summarized in Figure 1.

### 2.2. Data Sources and Variables

Patient demographics, liver disease etiology, and pre-LDT laboratory values were extracted from the electronic medical record. Serum AFP biomarker values were obtained as part of routine standard of care and reported at the time of HCC diagnosis. Radiographic assessment of HCC burden was obtained from the multidisciplinary HCC board reports prior to LDT. Time to progression (TTP) was evaluated from date of first-line LDT to primary endpoint of dropout due to HCC progression censoring for LT. Patients declined for continued LDT as a bridge to LT for reasons not attributable to HCC progression were censored on the date of the corresponding multidisciplinary HCC board decision. Patients remaining who remained active bridge to LT candidates under LT evaluation or on the LT waitlist were censored at the time of final data analysis.

### 2.3. Liver-Directed Therapy and Bridge to Transplant

First-line LDT modality was selected by the multidisciplinary HCC board. The board consisted of LT surgeons, hepatologists, and interventional radiologists with selection based upon performance status as well as characteristics related to size and location of the HCC. Institutional criteria for LDT were (a) Barcelona Clinic Liver Cancer Stage A/B, (b) Child-Pugh A/B, (c) non-resectable HCC, (d) without main portal vein thrombus or extrahepatic metastasis, (e) total bilirubin <4 mg/dL, (f) serum creatinine concentration <1.5 mg/dL, and (g) absent gross ascites by ultrasound or CT. First-line LDT included drug-eluting embolic transarterial chemoembolization (DEE-TACE), ^90^Yttrium transarterial radioembolization (^90^Y), or percutaneous microwave ablation (MWA). DEE-TACE was performed using 100–300 µm drug-eluting beads (LC Bead, BTG, England) loaded with 50 mg doxorubicin per vial. All vessels feeding the areas of tumor were treated prior to follow-up imaging. ^90^Y was performed as a 2-phase treatment, including a mapping angiogram with calculation of lung shunt fraction followed by ^90^Y glass microsphere infusion (TheraSphere; Boston Scientific, Marlborough, Massachusetts) [22]. Mapping angiography included vascular evaluation of the celiac artery, superior mesenteric artery, proper hepatic artery, and all feeding hepatic arteries to the areas of tumor. During treatment, all feeding vessels to areas of the tumor were treated with target radiation doses greater than 200 Gy. Ablation was performed using a high-powered, gas-cooled, multiple antenna-capable system (Neuwave Medical, Madison, WI, USA). Duration of treatment and power application were determined by the performing physician at the time of treatment and based on manufacturer guidelines with adjustments made for the tumor size or proximity to vulnerable structures.

The study period overlapped a change in the institutional algorithm for first-line treatment of HCC. Patients receiving first-line LDT in 2016 received DEE-TACE. From 2017 until study conclusion, the institutional algorithm was MWA for an ablatable index HCC < 3 cm with ^90^Y as first line for non-ablatable index HCC. Patients with contraindications for both MWA and ^90^Y received DEE-TACE as first-line LDT.

First-line response to LDT was evaluated as defined by the Response Evaluation Criteria in Solid Tumors modified for HCC (mRECIST) in all patients with follow-up imaging [23]. Assessment of mRECIST was modality-dependent with follow-up imaging at 30 days (DEE-TACE) or 60 days (^90^Y and MWA). In patients receiving sequential LDT to the index lesion without intermittent imaging follow-up, the mRECIST was evaluated following the final treatment in the sequence. Treatment track after follow-up imaging was dictated by the multidisciplinary HCC board and mapped to the following treatment tracks: (a) satisfactory response to treatment or successfully downstaged to within Milan Criteria with recurring 3 month surveillance until LT, (b) repeat treatment of the index HCC or non-index burden as a continued bridge to LT, (c) met study criteria for censoring for reasons not attributable to HCC progression beyond Milan Criteria, or (d) declared no longer a transplant candidate due to HCC progression or failure to downstage within Milan Criteria with referral for advanced stage treatment.

### 2.4. Biomarker Measurements

Blood specimens for biomarker analysis were obtained immediately prior to LDT on the day of procedure. Blood was collected into sodium citrate tubes and immediately processed to obtain cell-free plasma. AFP, AFP L3%, and DCP levels were then analyzed using the µTASWako i30 (FUJIFILM Wako Diagnostics, Mountain View, CA). Analyte pherograms were validated using Owlet software (FUJIFILM Wako Diagnostics, Mountain View, CA) by an instrument specialist blinded to the purpose of the study. Minimum detectable ranges were AFP > 0.3 ng/mL, AFP-L3 > 0.5%, and DCP > 0.10 ng/mL. Biomarker values at the minimum of detection were recorded as the minimum value.

### 2.5. Statistical Analysis

The central tendency for continuous variables was reported as the median and interquartile range. Categorical variables are summarized as the number of individuals matching the criteria and percentage with respect to the total cohort. Linear regression of continuous variables was performed in SAS JMP version 13.0 (SAS Institute, Cary, NC, USA) with log transformation and plotted with GraphPad Prism (San Diego, CA, USA) with coefficient of determination (R2). In outcomes analysis, only patients with an endpoint of transplantation or HCC progression were included in the analysis. Outcome analysis was performed using logistic regression of the continuous biomarker values followed by analysis for maximum area under the curve using the receiver operating characteristic (ROC) curve in SAS JMP. Kruskal–Wallis H test was used for nonparametric significance testing of continuous variables between multiple groups. Categorical variables were analyzed using Fisher’s Exact Test (binary variables) or Likelihood-ratio Chi-square (more than 2 levels). Multivariate analysis of factors associated with TTP was performed using the Cox proportional hazards model to identify hazard ratio [3] and 95% confidence interval. Univariate factors with a significance level < 0.100 were included in the multivariate model. Absolute AFP-L3 significantly correlated with total AFP and was therefore excluded from multivariate analysis (Supplementary Figure S1A).

## 3. Results

### 3.1. Cohort Baseline Overview, Response to First-Line LDT, and Primary Study Outcomes

The study cohort included 140 patients with recently diagnosed HCC scheduled to receive LDT. The cohort was predominantly Caucasian/White and male with a median age of 63 years (Table 1). Cohort medians for liver functional labs and Model of End-Stage Liver Disease (MELD-Na) score reflect well-compensated cirrhosis in early-stage HCC with Hepatitis C virus as the primary etiology of cirrhosis. The cohort was predominantly within Milan Criteria with a median index HCC 2.9 cm in diameter. Median AFP at diagnosis was 13 ng/mL, slightly above the normal range (8 ng/mL), with minimal change from diagnosis to pre-procedure (median AFP 12.9 ng/mL). Further, there was a significant correlation (*p* < 0.001, R2 0.88) between AFP measured in the serum at the time of diagnosis and AFP measured in the plasma pre-LDT (Supplementary Figure S1B). Median AFP-L3 (7% AFP-L3 fraction) and DCP (3.1 ng/mL) were both below the manufacturer-specified level for a positive test when utilized as an HCC surveillance assay (10% and 7.5 ng/mL, respectively).

DEE-TACE and 90Y were the most frequently utilized first-line LDT, with 73/120 (61%) patients with follow-up imaging having an objective first-line response (combined complete and partial response rate). Disease progression following first-line LDT was observed in 34/120 (28%) patients. Follow-up imaging was not available in 20/140 (14%) patients.

Cohort status toward the primary endpoint at the time of data analysis is summarized in Table 2. In the 140-patient study cohort, 41/140 (29%) were successfully bridged to LT, with 25/140 (18%) remaining active bridge to LT candidates. In the 34/140 (24%) study participants censored for survival analysis, the majority either declined LT listing with a minimum 1-year sustained complete radiographic response to LDT or were lost to follow-up post-treatment. In the 40/140 (29%) patients with HCC progression precluding continued LDT and LT waitlisting, the majority experienced growth of the index lesion or new intrahepatic HCC burden post-LDT resulting in progression beyond Milan Criteria for LT.

### 3.2. Biomarker Associations with HCC Progression, Threshold Levels

The established biomarker positive levels for the HCC surveillance assay are AFP > 20 ng/mL, AFP-L3 > 10%, and DCP > 7.5 ng/mL. When applied to pre-surgical HCC recurrence risk, threshold values range much higher with a wide variance for each biomarker across the literature. With the goal of assessing bridge to LT prognosis using biomarker expression at diagnosis, surveillance-relevant threshold values were prioritized. Surveillance thresholds were confirmed as setpoints using ROC analysis of the biomarker baseline with HCC progression risk in patients with LT or HCC progression at the primary endpoint (*n* = 81). Each biomarker was significantly associated with HCC progression risk (*p* < 0.001), including AFP at diagnosis as well as each individual biomarker assessed prior to first-line LDT (Table 3). ROC analysis for each biomarker revealed maximum area under the curve targets of 8 ng/mL for AFP, 13% for AFP-L3%, 2.5 ng/mL for absolute AFP-L3, and 6.1 ng/mL for DCP. For subsequent analysis, ROC values below surveillance recommendation were utilized at the recommendation (AFP > 20 ng/mL and DCP > 7.5 ng/mL), with the AFP-L3 fraction above surveillance recommendation rounded from 13% to > 15%.

### 3.3. Diversity in AFP, AFP-L3%, and DCP Profile in Treatment Naive, Bridge to LT Patients

**Figure 2:**
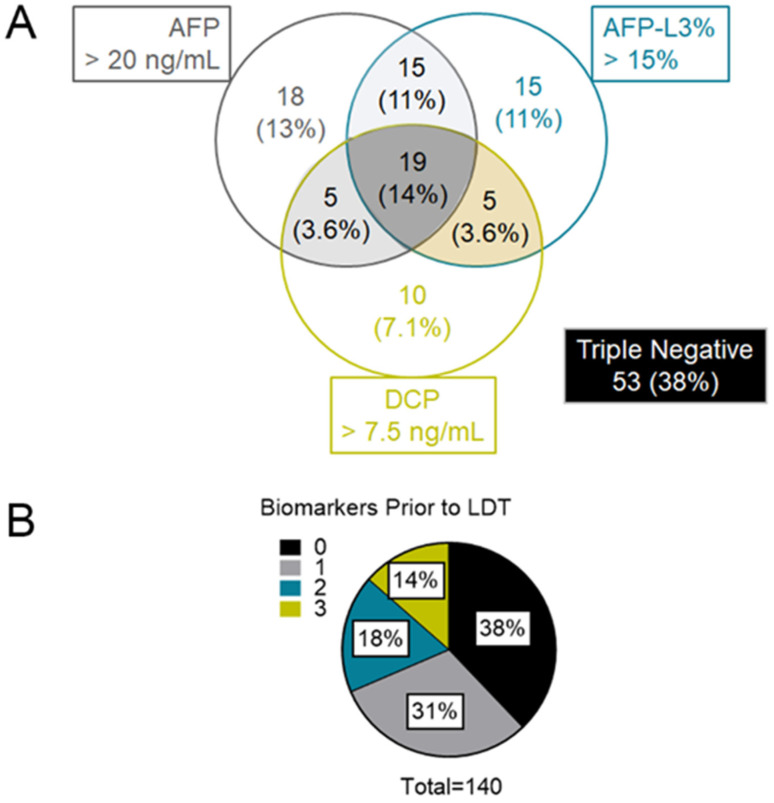
Biomarker Profile and Status Prior to LDT. (**A**) Venn diagram showing overlapping expression of the biomarkers by total number with percentage of the cohort in brackets. Biomarker triple negative patients are identified in the box next to the diagram. (**B**) Biomarker breakdown based on count of total biomarkers and labeled by percentage of the entire study cohort.

Biomarker profiles were then analyzed after converting continuous levels to binary positive/negative levels (Figure 2A). Analysis of the baseline cohort (*n* = 140) revealed that 53/140 (38%) patients were triple negative for the biomarkers prior to first-line LDT with the remaining cohort expressing at least one biomarker (Table 4). Triple negative patients had a smaller index tumor diameter (*p* = 0.003), less multifocal disease (*p* = 0.010), and an improved ORR (triple negative 62%, any biomarkers 46%). There was no difference in general demographics, etiology of cirrhosis, or preserved liver function based on the expression of any biomarker compared to triple negative.

Within the 87/140 (62%) total patients testing biomarker-positive, the expression patterns between AFP, AFP-L3, and DCP were spread across all possible phenotypes. The most prevalent expression pattern was biomarker triple positive (19/87 (22%)). Although AFP was the most frequently expressed biomarker (57/87 (66%)), there was substantial diversity in AFP phenotype with only 18/57 (32%) AFP positives expressing only the AFP biomarker. Conversely, 69/87 (79%) patients in the cohort expressed either AFP-L3% or DCP at baseline, with 30/87 (34%) patients expressing only AFP-L3% or DCP.

### 3.4. Associations between Biomarker Accumulation with Baseline Prognostic Factors and Response to LDT

With substantial diversity in biomarker profiles, the numerical accumulation of biomarkers was examined to increase the size of subgroups for subsequent analysis. The biomarker expression pattern decreased from triple negative to triple positive with 53/140 (38%) triple negative patients, 43/140 (31%) expressing one, 25/140 (18%) expressing two, and 19/140 (14%) expressing all three biomarkers (Figure 2B). The relationship between demographic variables as well as cirrhosis- and HCC-linked baseline factors was then investigated as the biomarker number accumulated (Table 4). Comparisons among biomarker expressing groups revealed no significant differences among general demographic factors or variables linked to the progression of cirrhosis with increasing biomarker expression. Notably, there was also no difference in baseline HCC burden or ORR across biomarker expression levels, although the frequency of multifocal disease and the size of the index HCC trended higher in the triple biomarker group.

### 3.5. LDT Baseline Factors, Biomarker Expression and Accumulation in Bridge to LT Prognosis

Univariate analysis was performed to analyze the role of treatment baseline factors as well as biomarker expression and accumulation on TTP after first-line LDT (Table 5). Baseline variables covering general demographics, cirrhosis background, and liver function were not associated with TTP. Radiographic HCC burden, a biomarker positive for AFP, AFP-L3, or DCP individually, and the total number of biomarkers were each associated with TTP in univariate analysis. The accumulation of biomarkers from 0 to 1 and from 1 to 2 decreased survival odds with an increase from 2 to 3 not reaching significance. Two approaches were used to incorporate biomarker data in the multivariate model. The first model utilized only the individual biomarkers (AFP, AFP-L3%, and DCP), while the second model utilized only the numerical accumulation of positive biomarkers. Since multifocal burden and index HCC diameter are incorporated in Milan Criteria, only Milan Criteria were utilized in the multivariate model to account for HCC radiographic burden.

Controlling for Milan Criteria, the AFP and DCP biomarkers at baseline were each associated with TTP. Baseline AFP had the higher HR of 4.6 with DCP having a 2.4 HR. The second multivariate model was controlled for Milan Criteria and utilized the accumulation of biomarkers grouped as triple negative (0 biomarkers), 1 biomarker, or 2–3 biomarkers based on the results of the univariate analysis. Baseline biomarker positives were associated with TTP, with the greatest HR of 4.8 with an increase from 1 to 2–3 biomarkers and a 3.3 HR from an increase of 0 to 1 biomarker.

### 3.6. Time to Progression by Biomarker Count and First-Line Complete Response to LDT

**Figure 3:**
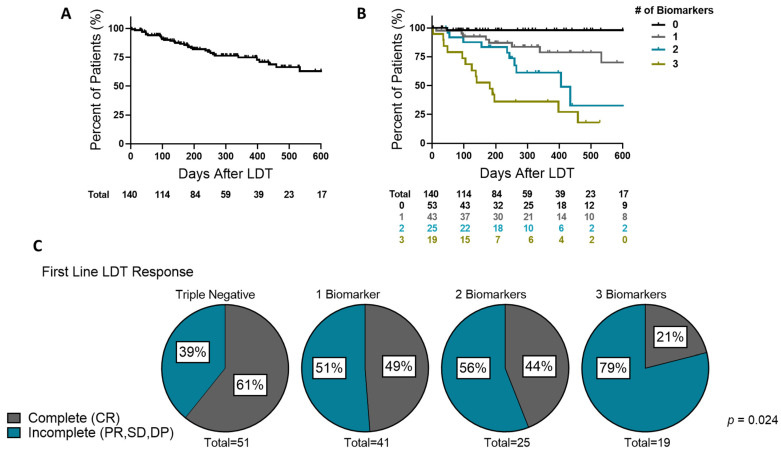
Time to Progression and First Line LDT Response by Biomarker Count. (**A**) Kaplan Meier curve of time to progression for the entire study cohort with total surviving patients at each time listed in the table below the graph. (**B**) Kaplan Meier curve of time to progression stratified by total biomarker count. (**C**) Percentage of patients with a complete re-sponse to first line LDT broken down by initial biomarker count. P value indicates significance in Chi Square Likelihood Ratio Test. Censored data in the Kaplan Meier curves is marked with a vertical upward dash.

Kaplan–Meier curves were then used to analyze time to progression following LDT associated with the biomarker baseline and examine the complete response rate (CRR) as a function of biomarker accumulation. CRR provides a well characterized, post-LDT prognostic measure associated with TTP. Reference points of 180 days post-LDT (corresponding to the 6-month minimum time point to receive waitlist MELD exception points to increase LT prioritization) and 275 days post-LDT (representing the center’s median time from first-line LDT to LT) were used. Survival rates for the cohort were 85% at 180 days and 76% at 275 days, with a median survival of 775 days (Figure 3A). Stratifying survival by the number of biomarkers at baseline resulted in significant survival outcomes, with median survival decreasing with an increasing number of biomarkers. Median survival from triple negative accumulating to triple positive was 994 (0), 799 (1), 406 (2), and 181 (3) days (Figure 3B). The 180-day survival percentages for triple negative (0), single (1), double (2), and triple positive (3) were 98%, 87%, 83%, and 53%. At the LDT to LT median of 275 days, survival percentages increasing from triple negative to triple positive were 98%, 84%, 61%, and 36%.

Initial analysis of ORR (complete or partial response) revealed a higher response rate in triple negative biomarker patients with a similar ORR profile among patients expressing one to all three biomarkers. The high survival rate in biomarker triple negative patients and rapid post-LDT progression rate in biomarker triple positive patients prompted the analysis of CRR (Figure 3C). Triple negative biomarker patients had a 63% CRR, which plummeted to 12% in biomarker triple positive patients.

## 4. Discussion

This study examined bridge to LT prognosis associated with baseline expression of the biomarkers AFP-L3 and DCP in combination with the clinical standard AFP. The study population received first-line LDT for HCC at a high-volume (60 LT/year with primary indication of HCC), short wait-time (median 275 days from first-line LDT to LT) center. The AFP level has prognostic implications throughout the bridge to LT timeline, including overall survival, post-LDT prognosis, presurgical recurrence risk, and post-LT surveillance [21,24,25,26]. In Japan and other parts of Asia, AFP is used in combination with AFP-L3% and DCP for HCC surveillance and the combined panel is studied in the context of overall, post-surgical, and recurrence-free survival outcomes [9,10,11,12,14,15,16,17].

With increased detection of early-stage disease due to HCC surveillance programs, AFP levels at diagnosis continue to historically decline. The median AFP at diagnosis for our center has declined annually from 34 ng/mL in 2016, to 15 ng/mL in 2017, and 8.4 ng/mL in 2018. Despite these trends in early-stage diagnosis and AFP biomarker levels, first-line ORR in bridge to LT remains highly variable in the literature (23%–94%) [27,28,29,30,31,32], with up to 25% of candidates experiencing HCC progression while awaiting LT [33,34,35]. With a potentially increasing incidence of AFP negativity and AFP <20 ng/mL, the ability to assess baseline prognosis using a single biomarker will become extremely limited. To address this limitation, AFP, AFP-L3%, and DCP were assessed in patients receiving LDT as a bridge to LT and utilized to assess prognosis based on combined biomarker expression. In a 4-year, 140-patient prospective study, positive expression of each biomarker individually was associated with the risk of HCC progression following LDT. This is in agreement with recent reviews showing AFP-L3% and DCP are each associated with aggressive HCC biology and overall prognosis [36,37], albeit using a wide range of threshold values. The positive thresholds used in this study are consistent with their use in HCC surveillance while also mirroring values recently validated for the pre-surgical assessment of HCC recurrence risk prior to LT [19,38,39].

At baseline, 62% of patients expressed at least one biomarker using the positive thresholds AFP > 20 ng/mL, AFP-L3% > 15%, and DCP > 7.5 ng/mL. Multivariate analysis controlling for Milan Criteria status revealed the baseline expression of two or more biomarkers was associated with the highest risk of bridge to LT tumor progression. In the multivariate analysis focused on the individual biomarkers, AFP > 20 ng/mL (HR 4.59) and DCP > 7.5 ng/mL (HR 2.37) were the only biomarkers associated with progression risk. A recent meta-analysis of multi-stage HCC patients receiving DEE-TACE demonstrated inferior progression-free survival/OS outcomes in patients positive for DCP prior to treatment [37]. A positive DCP biomarker prior to LT was also associated with recurrence risk post-LT [18].

The biomarker triple negative rate in this study (38%) mirrors the 40% rate observed at diagnosis in the 2600-patient Japanese BALAD cohort [40]. Biomarker triple negative patients (53/140) had lower disease burden at diagnosis, superior ORR and CRR profiles, and only one instance of HCC progression. Although first-line ORR and CRR have been associated with excellent bridge to LT and overall prognosis [41,42,43], these results support that biomarker triple negative patients are ideal bridge to LT candidates. Treatment to biomarker triple negative status could be the most critical factor associated with post-LT recurrence risk and warrants further prospective study.

The resulting profiles among biomarker-positive patients were diverse, with no single subtype having an incidence greater than 25%. Although AFP was the most frequent biomarker, only 18 of the 57 AFP-positive patients were also negative for AFP-L3% and DCP. This is consistent with other AFP, AFP-L3%, and DCP panel studies supporting biomarker diversity in AFP-positive HCC, which may have important implications in HCC biological aggressiveness and the optimal selection of a first-line LDT modality.

To address the diverse biomarker phenotypes, the numerical accumulation of biomarkers was utilized as recently described [19] and consistent with their use in the BALAD and GALAD prognosis/surveillance indices [18,44,45]. Biomarker accumulation was not associated with demographic or etiological factors. However, total HCC burden and the size of the index HCC trended higher with an increasing number of biomarkers, with only 62% of biomarker triple positive patients initially presenting within Milan Criteria. Patients beyond Milan Criteria at diagnosis and presenting with more than one biomarker (10/140) had a dismal post-LDT prognosis, with all patients experiencing HCC progression. This contrasts with triple negative or single biomarker patients, where 1-year post-LDT survival rates were 98% and 84%, respectively. These data align with several studies demonstrating an association between biomarker accumulation and poor recurrence-free and overall survival [13,16,18,19]. In 13 patients initially beyond Milan Criteria who experienced clinical disease progression, 10/13 had > 2 biomarkers prior to first-line LDT. The number of positive biomarkers could be critical in selecting patients beyond Milan Criteria amendable to downstaging with LDT.

This single-center study was designed to evaluate the role of baseline biomarker expression with post-LDT prognosis in the bridge to LT HCC. Limitations of the study are as follows. During the study period, our center shifted from DEE-TACE to ^90^Y as the first-line LDT modality for non-ablatable HCC. As a result, post-LDT biomarker analysis was excluded from the analysis due to modality-specific follow-up periods. Bias in the follow-up time for this multi-modality cohort and limitations in the intergroup cohort size precluded analysis. The first-line LDT modality may have important implications in ORR, retreatment approach, and ultimately HCC progression risk. Center-specific approaches to first-line LDT as a bridge to LT may limit the generalization of the results to other centers. Further, this study was conducted at a high-volume, short wait-time center, resulting in post-LDT surveillance and time to transplant periods that may not be translatable to other patient cohorts. This single center study will require multicenter validation to control for regional differences in HCC cohorts, multidisciplinary HCC board algorithms, and LT center-specific factors influencing the relationship between baseline biomarker profiles and bridge to LT outcomes.

## 5. Conclusions

This study highlights the value of the combined assessment of AFP, AFP-L3%, and DCP in treating naïve HCC undergoing LDT as a bridge to LT. Biomarker status may identify ideal candidates for LDT, with biomarker triple negative patients having improved ORR and superior TTP. In addition, the accumulation of biomarkers at baseline is a critical risk factor for 1-year HCC progression after LDT. A potential relationship between the post-LDT decrease in biomarkers to triple negative and progression-free/recurrence-free survival warrants further investigation. At baseline, the biomarker profile helps refine HCC progression risk, which may have important implications in selecting a first-line LDT modality and minimum waitlist period for LT. In conclusion, these results highlight the need for a more comprehensive HCC biomarker panel to determine ideal candidates as well as the optimal first-line LDT modality as a bridge to LT.

## Figures and Tables

**Table 1:** Bridge to Transplant Cohort Demographics, Baseline End-Stage Liver Disease Factors, and Tumor Characteristics.

General Demographics	Cohort (*n* = 140)
**Legal Sex, male (%)**	104 (74.2)
**Age at LDT, years (IQR)**	63 (59–66)
**Race, *n* (%)**	
African American/Black	38 (27.1)
Caucasian/White	93 (66.4)
Other	9 (6.4)
**Cirrhosis Background**	
**Etiology, *n* (%)**	
ALD	14 (10.0)
HCV	82 (58.6)
HCV/ALD	14 (10)
NASH	19 (13.6)
Other	11 (7.9)
**HCV Status, *n* (% of cohort)**	96 (68.6)
Viremic, *n* (% of HCV)	55 (57.3)
SVR, *n* (% of HCV)	41 (42.7)
**Liver Function Labs**	
Sodium, mM (IQR)	139 (137–140)
Creatinine, mg/dL (IQR)	0.9 (0.8–1.1)
Bilirubin, mg/dL (IQR)	1.1 (0.6–1.6)
INR, ratio (IQR)	1.1 (1.0–1.2)
MELD-Na, score (IQR)	11 (8–13)
Albumin, g/dL (IQR)	3.4 (3.7–2.9)
**Radiographic HCC Burden**	
Multifocal, *n* (%)	30 (21.4)
Largest lesion, cm (IQR)	2.9 (2.3–3.8)
Milan Criteria, *n* (%)	115 (82.1)
**Circulating HCC Biomarkers**	
Serum AFP (diagnosis), ng/mL (IQR)	13 (5.1–79)
AFP (pretreatment), ng/mL (IQR)	12.9 (3.7–81.2)
AFP-L3 Fraction (pretreatment), % (IQR)	11.1 (5.4–22.3)
Absolute AFP-L3 (pretreatment), ng/mL (IQR)	1.4 (0.4–14.6)
DCP (pretreatment), ng/mL (IQR)	3.1 (0.8–10)
**Liver-Directed Therapy**	
**First-Line Treatment, date (range)**	8/30/16–10/16/20
**Treatments to Endpoint, median (IQR)**	2 (1–2)
**First-Line Modality, *n* (%)**	
DEE-TACE	54 (38.6)
^90^Y	54 (38.6)
MWA	32 (22.9)
**mRECIST**	
**Response Level, *n* (%)**	
Complete	66 (47.1)
Partial	19 (13.6)
Stable	13 (9.3)
Progression	38 (27.1)
No Follow-up Imaging	4 (2.9)

Abbreviations: End-Stage Liver Disease (ESLD), Hepatocellular Carcinoma (HCC), Interquartile Range (IQR), Alpha-Fetoprotein (AFP), *Lens culinaris* Agglutinin-Reactive Alpha-Fetoprotein (AFP-L3), Des-Gamma-Carboxy Prothrombin (DCP), Drug-Eluting Embolic Transarterial Chemoembolization (DEE-TACE), Yittrium-90 Radioembolization (^90^Y), Microwave Ablation (MWA), Modified Response Evaluation Criteria in Solid Tumors (mRECIST), Liver-Directed Therapy (LDT), Interquartile Range (IQR), Alcoholic Liver Disease (ALD), Hepatitis C (HCV), Non-Alcoholic Liver Disease (NASH), Sustained Virologic Response (SVR), International Normalized Ratio (INR), Model of End-Stage Liver Disease (MELD-Na).

**Table 2:** Bridge to Transplant Cohort Outcomes.

Status at Data Analysis, *n* (% of total)	Cohort (*n* = 140)
Active	25 (17.9)
Censored	34 (24.3)
Bridged to Liver Transplant	41 (29.3)
Tumor Progression	40 (28.6)
**Censoring Event, *n* (% of subgroup)**	
Declined Listing with Sustained Complete Response > 1 yr	12 (35.3)
Lost to Follow-Up	13 (38.2)
Deceased	5 (14.7)
Other	4 (11.8)
**Cause of Tumor Progression, *n* (% of subgroup)**	
Progression Beyond Milan Criteria	23 (57.5)
Unable to Downstage Within Milan	5 (12.5)
AFP Persistently > or Increasing to > 1000 ng/mL	12 (30)

Abbreviations: Alpha-Fetoprotein (AFP).

**Table 3:** Univariate Analysis of AFP, AFP-L3%, and DCP with Bridge to Transplant Outcome of HCC Progression after Liver-Directed Therapy.

	Primary Endpoint (*n* = 81)		
Circulating HCC Biomarkers	*p*-Value	Max AUC	OR (95% CI)
Baseline Serum AFP, ng/mL	<0.001	13 ng/mL	
Elevated Serum AFP, > vs. ≤ 20 ng/mL	<0.001		5.06 (1.96–13.08)
Pretreatment AFP, ng/mL	0.003	8.3 ng/mL	
Elevated Pre-Procedure AFP, > vs. ≤ 20 ng/mL	<0.001		5.03 (1.96–12.91)
Pretreatment AFP-L3 Fraction, (%)	0.0154	12.6%	
Elevated Pre-Procedure AFP-L3%, > vs. ≤ 15%	0.005		3.63 (1.44–9.13)
Absolute AFP-L3, ng/mL	0.007	2.5 ng/mL	
Elevated Pre-Procedure AFP-L3, > vs. ≤ 2.7 ng/mL	<0.001		10.91 (3.86–30.80)
Des-gamma-carboxy prothrombin, ng/mL	<0.001	6.1 ng/mL	
Elevated Pre-Procedure DCP, > vs. ≤ 7.5 ng/mL	<0.001		8.75 (2.99–25.57)

Abbreviations: Receiver Operating Characteristic (ROC), Alpha-Fetoprotein (AFP), *Lens culinaris* Agglutinin-Reactive Alpha-Fetoprotein (AFP-L3), Des-Gamma-Carboxy Prothrombin (DCP), Hepatocellular Carcinoma (HCC), Area Under the Curve (AUC), Odds Ratio (OR), 95% Confidence Interval (95% CI).

**Table 4:** Baseline Factors Stratified by Biomarker Status.

	Cohort *n* = 140			Biomarker Positive Cohort *n* = 87			
General Demographics	Triple Negative (*n* = 53)	Any Biomarkers (*n* = 87)	*p* Value	1 Biomarker (*n* = 43)	2 Biomarkers (*n* = 25)	3 Biomarkers (*n* = 19)	*p* Value
Legal Sex, male (%)	41 (77)	63 (72)	0.556	31 (72)	17 (68)	15 (72)	0.723
Age at LDT, years (IQR)	64 (60–66)	62 (59–67)	0.486	62 (58–66)	62 (59–67)	64 (60–67)	0.528
Race, *n* Caucasian (%)	36 (68)	57 (66)	0.770	29 (67)	18 (72)	10 (53)	0.389
**Cirrhosis Background**							
Etiology, *n* HCV (%)	34 (64)	62 (71)	0.456	30 (70)	18 (72)	14 (74)	0.947
**HCV Status, *n* (% of subgroup)**							
Viremic, *n* (% of HCV)	16 (47)	39 (63)	0.195	21 (70)	8 (44)	10 (71)	0.162
**Liver Function Labs**							
Sodium, mM (IQR)	138 (137–140)	139 (137–141)	0.550	138 (137–140)	139 (136–142)	140 (137–141)	0.588
Creatinine, mg/dL (IQR)	0.9 (0.8–1.1)	0.9 (0.8–1.1)	0.557	0.9 (0.8–1.1)	0.8 (0.8–0.9)	0.9 (0.8–1.4)	0.334
Bilirubin, mg/dL (IQR)	1.1 (0.7–1.8)	1.1 (0.6–1.5)	0.278	0.8 (0.5–1.5)	1.1 (0.6–1.6)	1.2 (0.8–1.8)	0.229
INR, ratio (IQR)	1.1 (1.0–1.3)	1.1 (1.0–1.2)	0.970	1.1 (1.0–1.2)	1.1 (1.0–1.3)	1.1 (1.1–1.2)	0.679
MELD-Na, score (IQR)	11 (8–13)	11 (8–14)	0.704	11 (8–13)	10 (7–15)	11 (10–14)	0.549
Albumin, g/dL (IQR)	3.3 (3.0–3.7)	3.4 (2.9–3.7)	0.776	3.4 (2.9–3.7)	3.5 (2.9–3.8)	3.2 (2.9–3.6)	0.756
**Radiographic HCC Burden**							
Multifocal, *n* (%)	5 (9)	25 (29)	0.010	12 (28)	5 (20)	8 (42)	0.272
Largest Lesion, cm (IQR)	2.6 (2.1–3.3)	3.2 (2.4–4.1)	0.003	3.0 (2.3–4.0)	2.9 (2.5–4.0)	3.8 (2.8–4.8)	0.105
Milan Criteria, *n* (%)	48 (91)	67 (77)	0.067	33 (77)	22 (88)	12 (63)	0.149
**Liver-Directed Therapy**							
**First-Line Modality, *n* (%)**			0.134				0.993
DEE-TACE	23 (43)	31 (36)		15 (35)	9 (36)	7 (37)	
^90^Y	15 (28)	39 (45)		19 (44)	11 (44)	9 (47)	
MWA	15 (28)	17 (19)		9 (21)	5 (20)	3 (16)	
**mRECIST**							
**Response Level, *n* (%)**			0.011				0.799
Objective Response (CR/PR)	39 (74)	46 (53)		23 (53)	14 (56)	9 (47)	
Non-Objective Response (SD/DP)	12 (23)	39 (45)		18 (42)	11 (44)	10 (53)	
No Follow-up Imaging	2 (3.8)	2 (2.3)		2 (4.7)	0 (0)	0 (0)	

Abbreviations: Hepatocellular Carcinoma (HCC), Liver-Directed Therapy (LDT), Interquartile Range (IQR), Hepatitis C (HCV), International Normalized Ratio (INR), Model of End-Stage Liver Disease (MELD-Na), Drug-Eluting Embolic Transarterial Chemoembolization (DEE-TACE), Yittrium-90 Radioembolization (^90^Y), Microwave Ablation (MWA), Complete Response (CR), Partial Response (PR), Stable Disease (SD), Disease Progression (DP). Continuous variables analyzed using Mann–Whitney. Categorical binary variables analyzed by two-way Fisher’s Exact Test. Categorical variables with more than two levels analyzed using Chi Square.

**Table 5:** Univariate Analysis of AFP, AFP-L3%, and DCP with Bridge to Transplant Outcome of HCC Progression after Liver-Directed Therapy.

Cohort (*n* = 140)	Univariate		Multivariate Model 1		Multivariate Model 2	
General Demographics	*p* Value	HR (95%CI)	*p* Value	HR (95%CI)	*p* Value	HR (95%CI)
Legal Sex, male vs. female	0.230					
Age at LDT, years (IQR)	0.253					
Race, Caucasian/White vs. Other	0.948					
**Cirrhosis Background**						
**Etiology, HCV vs. Other**	0.299					
**HCV Status**	0.574					
Viremic vs. SVR	0.866					
Viremic vs. non-HCV	0.304					
SVR vs. non-HCV	0.433					
**Liver Function Labs**						
Sodium, mM (IQR)	0.871					
Creatinine, mg/dL (IQR)	0.786					
Bilirubin, mg/dL (IQR)	0.907					
INR, ratio (IQR)	0.946					
MELD-Na, score (IQR)	0.828					
Albumin, g/dL (IQR)	0.277					
**Radiographic HCC Burden**						
Multifocal vs. Unifocal	0.015	2.46 (1.21–4.82)				
Largest Lesion, units of 1 cm	< 0.001	1.45 (1.24–1.67)				
Outside vs. Within Milan Criteria	< 0.001	3.67 (1.77–7.35)	0.044	2.21 (1.02–4.67)	0.015	2.51 (1.21–5.06)
**Circulating HCC Biomarkers**						
Pre-Procedure AFP, > vs. ≤20 ng/mL	< 0.001	6.56 (3.07–15.70)	<0.001	4.59 (2.02–11.56)		
Pre-Procedure AFP-L3%, > vs. ≤15%	< 0.001	3.18 (1.66–6.33)	0.073	1.90 (0.94–3.95)		
Pre-Procedure DCP, > vs. ≤7.5 ng/mL	< 0.001	4.54 (2.39–8.82)	0.023	2.37 (1.13–5.00)		
Positive biomarkers	< 0.001				<0.001	
0 vs. 1	0.024	3.80 (1.18–16.83)				
1 vs. 2	0.010	3.46 (1.35–9.28)				
2 vs. 3	0.055	2.18 (0.98–4.96)				
0 vs. 1					0.044	3.34 (1.03–14.85)
1 vs. 2–3					<0.001	4.80 (2.18–11.78)
**Liver-Directed Therapy**						
**First-Line Modality, *n* (%)**	0.139					
DEE-TACE vs. ^90^Y	0.881					
DEE-TACE vs. MWA	0.074					
^90^Y vs. MWA	0.067					

Abbreviations: Hazard Ratio [3], 95% Confidence Interval (95% CI), Liver-Directed Therapy (LDT), Interquartile Range (IQR), Hepatitis C (HCV), Sustained Virologic Response (SVR), International Normalized Ratio (INR), Model of End-Stage Liver Disease (MELD-Na), Alpha-Fetoprotein (AFP), *Lens culinaris* Agglutinin-Reactive Alpha-Fetoprotein (AFP-L3), Des-Gamma-Carboxy Prothrombin (DCP), Drug-Eluting Embolic Transarterial Chemoembolization (DEE-TACE), Yittrium-90 Radioembolization (^90^Y), Microwave Ablation (MWA).

## Data Availability

The datasets generated during and/or analyzed during the current study are available from the corresponding author on reasonable request.

## Supplementary Materials

The following are available online at https://www.mdpi.com/article/10.3390/cancers13194765/s1, Figure S1: Correlations between Absolute AFP-L3 and Plasma AFP and Serum and Plasma AFP Values.
