# Supplementary material for: Baseline Alpha-Fetoprotein, Alpha-Fetoprotein-L3, and Des-Gamma-Carboxy Prothrombin Biomarker Status in Bridge to Liver Transplant Outcomes for Hepatocellular Carcinoma
Source: Cancers (Basel). 2021 Sep 23;13(19):4765. doi: 10.3390/cancers13194765 (PMC8507524; doi:10.3390/cancers13194765)
Supplement: Supplementary file 1 [file cancers-13-04765-s001.zip › cancers-1359506-supplementary.pdf]

Article

Baseline Alpha-Fetoprotein, Alpha-Fetoprotein-L3, and Des-Gamma-Carboxy Prothrombin Biomarker Status in Bridge to Liver Transplant Outcomes for Hepatocellular Carcinoma

Kelley G. Núñez [1], Tyler Sandow [2], Daniel Fort [3], Jai Patel [1], Mina Hibino [1], Ian Carmody [4], Ari J. Cohen [1,4,5] and Paul Thevenot [1,*]

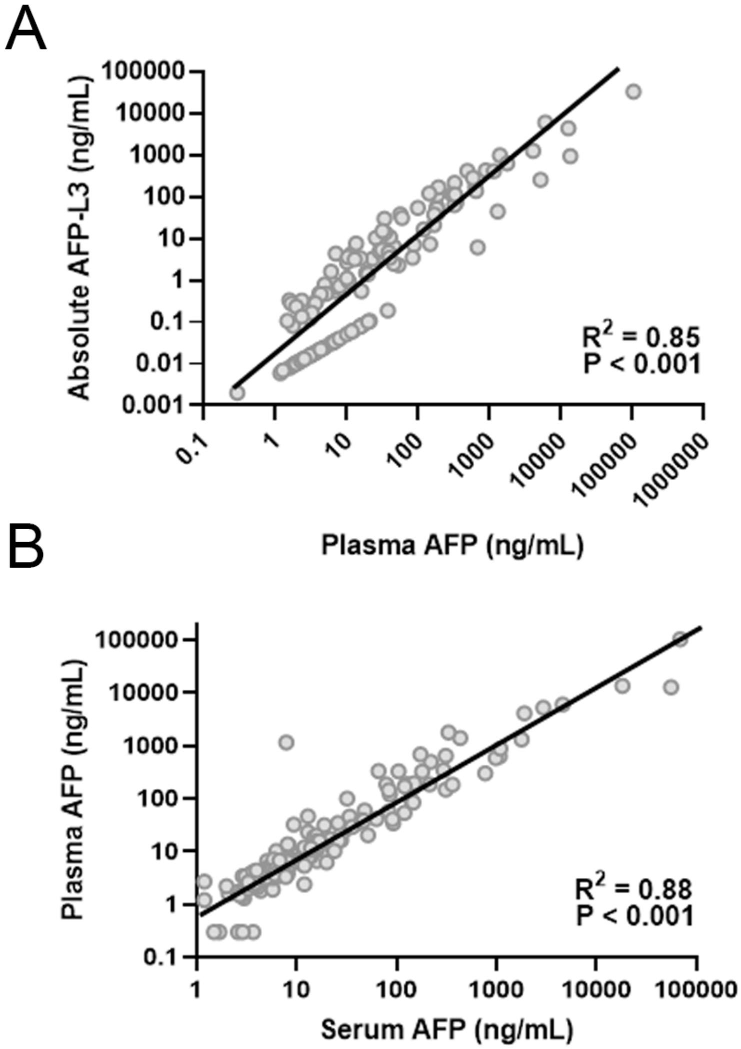

Figure S1. Correlations between Absolute AFP-L3 and Plasma AFP and Serum and Plasma AFP Values. **(A)** Absolute AFP-L3 values correlated with plasma AFP determined on the day of liver-directed therapy. **(B)** Serum AFP values determined at the time of hepatocellular carcinoma diagnosis correlated with plasma AFP values assessed on the day of liver-directed therapy.
